# CDKN2B antisense RNA 1 expression alleviates idiopathic pulmonary fibrosis by functioning as a competing endogenouse RNA through the miR-199a-5p/Sestrin-2 axis

**DOI:** 10.1080/21655979.2022.2044252

**Published:** 2022-03-16

**Authors:** Mei Yang, Egao Yin, Yiheng Xu, Yongjun Liu, Ting Li, Zhaoxing Dong, Wenlin Tai

**Affiliations:** a Department of Respiration, The Sencond Affiliated Hospital of Kunming Medical University, Kunming, Yunnan, China; b Department of Respiratory and Critical Care, Affiliated Hospital of Yunnan University, Kunming, Yunnan, China; c Department of Clinical Laboratory, Yunnan Molecular Diagnostic Center, the Sencond Affiliated Hospital of Kunming Medical University, Kunming, Yunnan, China; d Department of Respiration, Hwa Mei Hospital, University of Chinese Academy of Sciences, Ningbo, Zhejiang, China; e Department of Respiration, Ningbo Institute of Life and Health Industry, University of Chinese Academy of Sciences, Ningbo, Zhejiang, China

**Keywords:** CDKN2B-AS1, IPF, miR-199a-5p, SESN2, autophagy

## Abstract

Idiopathic pulmonary fibrosis (IPF) is an idiopathic interstitial lung disease. At present, the pathogenesis of IPF has not been fully elucidated, which has affected the development of effective treatment methods. Here, we explored the function and potential mechanism of long noncoding RNA (lncRNA) CDKN2B antisense RNA 1 (CDKN2B-AS1) in IPF.Transforming growth factor-β (TGF-β) and bleomycin (BLM) were used to induce IPF in cells and animal models. Real Time quantitative Polymerase Chain Reaction (RT-qPCR) showed the expression of CDKN2B-AS1, miR-199a-5p and Sestrin-2 (SESN2) in cells and tissues. The double luciferase reporter gene assay confirmed the targeting relationship among CDKN2B-AS1, miR-199a-5p, and SESN2. Related protein levels were detected by Western blot combined with Cell Counting Kit-8 (CCK-8), wound healing, and flow cytometry to analyze cell proliferation, migration, and apoptosis. The pathological characteristics of mouse lung tissue were determined by Hematoxylin-eosin (HE) and Masson staining. We found that the expression of CDKN2B-AS1 was decreased in TGF-β-treated cells and BLM-treated mice. Overexpression of CDKN2B-AS1 inhibited cell proliferation and migration, promoted apoptosis, decreased the expression of fibrosis-related proteins and promoted autophagy. In addition, overexpression of CDKN2B-AS1 alleviated pulmonary fibrosis in BLM-treated mice. Mechanistically, CDKN2B-AS1 acts as a miR-199a-5p sponge to regulate SESN2 expression. Our results indicate the importance of the CDKN2B-AS1/miR-199a-5p/SESN2 axis.

## Graphical abstract

**Figure:**
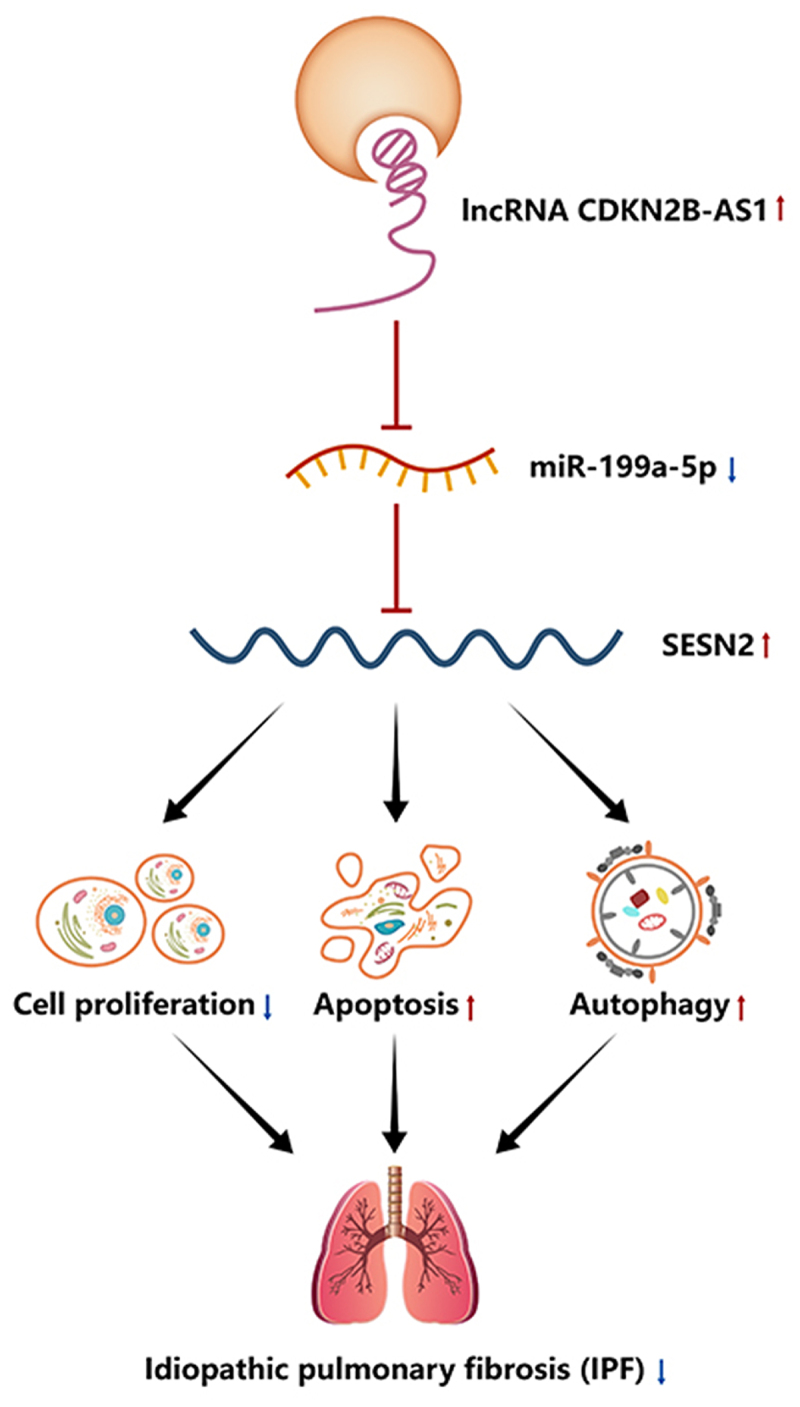

## Introduction

Idiopathic pulmonary fibrosis (IPF) is a serious fibroproliferative lung disease characterized by the accumulation of lung fibroblasts and extracellular matrix (ECM) deposition. Approximately thousands of new patients are diagnosed with IPF every year, and the median survival period after IPF diagnosis is 3 to 5 years, with a 5-year survival rate of only 20% [1,2]. Although the new antifibrotic drugs pirfenidone and nintedanib are approved for IPF treatment, neither is capable of stabilizing or improving lung function status in patients [3]. Therefore, the development of biomarkers associated with both the diagnosis and progression of IPF might improve personalized care, observations and treatment decisions and could broadly affect the approach to the early diagnosis of IPF [4].

Long noncoding RNAs (lncRNAs) are defined as transcripts that are greater than 200 nucleotides in length and do not encode proteins. lncRNAs have rich biological functions and are widely involved in various physiological processes in organisms [5]. Their fine molecular regulation mechanisms have been widely revealed to be involved in cell apoptosis, proliferation and other biological processes. Studies have shown that lncRNAs play significant roles in the occurrence and evolution of diseases [6,7]. LncRNA CDKN2B-AS1, as a potential lncRNA, has been demonstrated to be abnormally expressed in various malignant tumors [8], including gastric cancer, lung cancer, and breast cancer, with implications in the proliferation and migration of tumor cells [9,10]. Moreover, CDKN2B-AS1 is related to many nonmalignant diseases [11,12]. Therefore, CDKN2B-AS1 is a therapeutic target and prognostic biomarker of human diseases. In addition, studies have shown that, through high-throughput sequencing and bioinformatics analysis, the expression of CDKN2B-AS1 in peripheral blood of IPF patients is significantly reduced compared with that of healthy controls [13]. However, the role of CDKN2B-AS1 in IPF is not clear. We thoroughly investigated the functions and associated mechanisms of CDKN2B-AS1 in IPF.

Matrix overremodeling is a dynamic, complex pathological process in IPF. In this process, preinflammation, mitochondrial reactive oxygen species (ROS) generation, ECM accumulation and fibroblast foci formation occur. Normal lung tissues are replaced by fibrotic tissue, thus causing decreased exchange and impaired pulmonary homeostasis [14]. Many fibroblasts are activated to form lesions, and inflammatory cells infiltrate and release multitudinous inflammatory factors [15], which irreversibly abolish the normal physiological functions of lung tissue. Severe pulmonary fibrosis causes difficulty breathing, respiratory failure or even death. Consequently, suppressing the activation of fibroblasts and maintaining lung cell homeostasis are key to the treatment of IPF.

Autophagy is a self-consumption catabolic process by which damaged proteins and organelles are lysosomally degraded and plays a crucial role in the maintenance of cellular homeostasis, especially during cell starvation or other stress stimulation [16]. Increased ratios of LC3-II/I and LC3-II/p62 are believed to be hallmarks of autophagy generation. In IPF research, the role of autophagy has received attention [17]. Most factors that promote pulmonary fibrosis, such as oxidative stress, endoplasmic reticulum stress, and hypoxia, can induce autophagy [18]. Autophagic flow is reduced when lung tissue exhibits fibrotic lesions in bleomycin (BLM) and TGF-β-induced IPF mouse models, as well as in a TGF-β-mediated fibroblast-to-myofibroblast differentiation (FMD) in vitro model [19]. Hill et al. [20] found that autophagy inhibition induces the epithelial–mesenchymal transition (EMT) of alveolar epithelial cells and contributes to fibrosis. In addition to regulating cell survival, apoptosis, and EMT and FMD in fibroblasts, autophagy is involved in collagen degradation.

This study aimed to clarify the expression characteristics, potential roles and mechanisms of CDKN2B-AS1 in IPF. We hypothesized that CDKN2B-AS1 was decreased in TGF-β-treated cells and BLM-treated mice. Overexpression of CDKN2B-AS1 can inhibited cell proliferation and migration, promoted cell apoptosis, reduced the expression of fibrosis-related proteins, and promoted cell autophagy. We also hypothesized that CDKN2B-AS1 acts as a Mir-199a-5p sponge to regulate SESN2 expression. Our work may reveal an important role of CDKN2B-AS1 in alleviating the development of IPF, which may provide a new promising molecular target for the treatment of IPF.

## Materials and methods

### Cell culture

Human fetal lung fibroblast 1 (HFL-1) cells were purchased from American Type Culture Collection (ATCC, USA) and cultured in Ham’s F-12 K (Kaighn’s) medium (Gibco, USA) containing 10% fetal bovine serum (FBS, Gibco, USA) and 1% penicillin–streptomycin (Sigma–Aldrich, USA) at 37°C in a 5% CO_2_ environment. The model cell group cultured at 37°C for 8–12 hours and then incubated with 10 ng/ml human recombinant TGF-β1 (Sigma-Aldrich, USA) for 24 h.

### Cell transfection

HFL-1 cells were seeded into 6-well plates at 1 × 10^5^ cells/well. When the cell density was approximately 90%, Lipofectamine 3000 (Thermo Fisher Scientific, USA) was applied to transfect the CDKN2B-AS1 overexpression plasmid, miR-199a-5p mimic/inhibitor, or SESN2 inhibitor (Oligobio, China) into the cells according to the manufacturer’s instructions, and the control group was transfected with an empty plasmid. Total RNA or total protein were extracted 48 hours after transfection to complete the detection of subsequent experimental indicators.

### RT–qPCR

TRIzol (Thermo Fisher Scientific, USA) was used to extract total RNA from cells and tissues, and cDNA was synthesized with a reverse transcription kit (Promega, USA). SYBR Green Master Mix (Thermo Fisher Scientific, USA) was used to measure the expression levels of lncRNAs, miRNAs and mRNAs. Initial activation was performed at 95°C for 2 min, followed by 40 cycles of denaturation at 95°C for 15s, annealing at 60°C for 25s, and extension at 72°C for 60s. GAPDH was used as an endogenous control for lncRNAs and mRNAs, and U6 was used as an endogenous control for miRNAs. The 2^−ΔΔCT^ method was used to analyze relative expression among samples [21]. The primer sequences were (5’-3’) CDKN2B-AS1 Forward (F): TCATCATCATCATCATCATC and Reverse (R): TGCTTCTGTCTCTTCATA ′; miR-199a-5p F: GCCCAGTGTTCAGACTACCTG and R: GTGCAGGGTCCGAGGTATTC; GAPDH F ACAACTTTGGTATCGTGGAAGG and R: GCCATCACGCCACAGTTTC; and U6 F: GGGCAGGAAGAGGGCCTAT and R: TATGGCTAGCATGACTGGT.

### Cell Counting Kit-8 (CCK-8)

The analysis was performed with a Cell Counting Kit-8 (MedChem Express, USA) according to the manufacturer’s protocol. Cells were cultured in 96-well plates at 10^3^ cells/well. After 24 h of incubation, 10 μL CCK-8 solution was added to the medium, and the cells were incubated at 37°C for 2 h. The absorbance was measured at 450 nm on a microplate reader (Mairui, China), and the cell proliferation rate was calculated [22,23].

### Flow cytometry

Cell apoptosis was analyzed by flow cytometry (Beckman Coulter, USA). Briefly, cells in the logarithmic growth phase were inoculated into a 96-well plate. After overnight incubation, the cells were washed and resuspended in binding buffer. The cells were incubated with Annexin V-FITC (Procell, China) for 15 min at room temperature in the dark. Then, PI staining solution was added, and the binding liquid was replenished [24].

### Western blot

RIPA buffer (Thermo Fisher Scientific, USA) was used to extract the total protein from the tissues and cells in each group, and a BCA kit (Beytime, China) was used to detect the protein concentration and purity. The proteins were separated by 10% SDS–polyacrylamide gel electrophoresis (PAGE), and the protein bands were transferred to polyvinylidene difluoride (PVDF) membranes. After blocking in TBST solution containing 5% skimmed milk for 2 h at room temperature, SESN2, α-SMA, COL1A1, COL3A1, LC3I, LC3II, and p62 primary antibodies (1:1000, Cell Signaling Technology, USA) were added and incubated with the cells overnight at 4°C. Next, horseradish peroxidase-coupled secondary antibody (1:5000, Cell Signaling Technology, USA) was added, and the cells were incubated at room temperature for 2 h. The ECL chemiluminescent reagent (Beytime, China) was used to visualize the bands, grayscale analysis was performed with ImageJ software, and protein values were calculated in combination with GAPDH expression [25].

### Wound healing

Cells were seeded into a 6-well plate at 10^6^ cells/well and cultured until they were fully confluent. A 20 μL pipette tip was used to make wounds in the cell monolayer. After 48 h, the cells were washed to remove nonadhered cells, and images were captured to compare the widths of the scratches and evaluate cell migration.

### Dual-luciferase reporter gene

The ‘starBase’ biological information database was used to predict the targeted binding sequence of miR-199a-5p and CDKN2B-AS1/SESN2, and the binding sequence was inserted into the pMIR-report plasmid (Ambion, USA). A pMIR-report-CDKN2B-AS1/SESN2-WT wild-type plasmid and pMIR-report -CDKN2B-AS1/SESN2-MUT binding site-mutant plasmid were constructed. Cells were seeded into 24-well cell culture plates and cotransfected with the wt/mut plasmid and miR-21 mimic/NC mimic after 12 hours. After 48 h, luciferase activity was detected with the dual luciferase reporter gene kit.

### Animal model

Male C57BL/6 mice of 8–10 weeks old were obtained from the Experimental Animal Center of Kunming Medical University. The pulmonary fibrosis model was established by the tracheal instillation of bleomycin, with 6–8 mice in each group. The model group was injected with bleomycin (BLM, 5 mg/kg), and the control group was injected with the same amount of normal saline [26,27]. To observe the therapeutic effect of CDKN2B-AS1 on idiopathic pulmonary fibrosis in mice, the overexpressed CDKN2B-AS1 plasmid (1 nmol/mouse) was injected through the tail vein on day 10 after BLM stimulation, and then every 4 days after that, the mice were sacrificed on day 28. The mouse experiment was approved by the Animal Ethics Committee of Kunming Medical University (NO. Kmmu2021744) and carried out in accordance with the ‘Guidelines for the Care and Use of Laboratory Animals’ published by the National Institutes of Health.

### Hematoxylin–eosin (HE) and Masson staining

Mouse lung tissue samples were fixed with 4% paraformaldehyde (PFA, DingGuo, China), dehydrated in an ethanol gradient, embedded in paraffin and sectioned (5 μm). HE and Masson staining were separately performed. HE staining involved dewaxing, hematoxylin staining, hydrochloric acid ethanol treatment, ammonia treatment, eosin staining, ethanol gradient dehydration, xylene penetration, and neutral resin mounting. Morphological changes in lung tissue were observed under a microscope, and images were collected. Masson staining involved dewaxing, hematoxylin staining, Ponceau staining after washing, glacial acetic acid treatment, molybdophosphoric acid treatment, aniline blue staining, alcohol dehydration, xylene penetration, and neutral resin mounting. Fibrosis degrees were observed under a microscope, and images were collected.

### Statistical analysis

All experimental data were processed using GraphPad Prism 5, and results are expressed as the mean ± standard deviation (SD). Comparisons of two groups were performed by two-tailed Student’s t tests, and comparisons of multiple groups were performed by one-way analysis of variance (ANOVA). Result with p < 0.05 were considered statistically significant.

## Results

We suspect that the inhibition of IPF by CDKN2B-AS1 acts through a ceRNA mechanism and is related to the activation of autophagy. In a TGF-β-induced IPF cell model, we performed proliferation, Wound healing, and apoptosis assays to evaluate the biological behavior of cells, and to detect the expression of pulmonary fibrosis-related proteins and autophagy-related proteins. In the bleomycin-induced IPF animal model, HE and Masson staining were used to observe the effect of CDKN2B-AS1 on pulmonary fibrosis in mice. We found that CDKN2B-AS1 inhibited the proliferation of pulmonary fibrotic cells by regulating the miR-199a-5p/SESN2 axis, and promoted the occurrence of apoptosis and activated autophagy, and finally alleviated the occurrence of pulmonary fibrosis in mice.

### CDKN2B-AS1 decreased during fibrosis in vivo and in vitro

**Figure 1.**
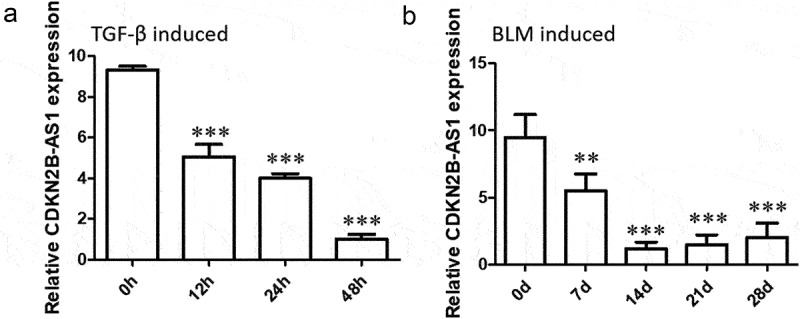
CDKN2B-AS1 expression is reduced in mouse and cell fibrosis models. a: RT–qPCR revealed the levels of CDKN2B-AS1 in cells treated with TGF-β at 0, 12, 24, and 48 hours. ***p < 0.001 compared with 0 h. b: RT–qPCR revealed the levels of CDKN2B-AS1 in mice treated with BLM on days 0, 7, 14, 21, and 28 days (n = 5). ***p < 0.001 compared with 0 d. h: hour, d: day.

To explore the role of CDKN2B-AS1 in pulmonary fibrosis, we established a TGF-β-induced fibrosis cell model and a BLM-induced pulmonary fibrosis mouse model. RT–qPCR was used to analyze the expression of CDKN2B-AS1 in cells and tissues. The results showed that the expression of CDKN2B-AS1 in HFL-1 cells induced by TGF-β gradually decreased over time and reached a minimum at 48 h (Figure 1a). The expression of CDKN2B-AS1 in BLM-induced pulmonary fibrosis mice was consistent with the results obtained in cells, with CDKN2B-AS1 gradually decreasing over time and reaching a low point at 14 d (Figure 1b). These results indicate that CDKN2B-AS1 is decreased in lung fibrosis in vivo and in vitro.

### CDKN2B-AS1 overexpression inhibits fibroblast activation

**Figure 2.**
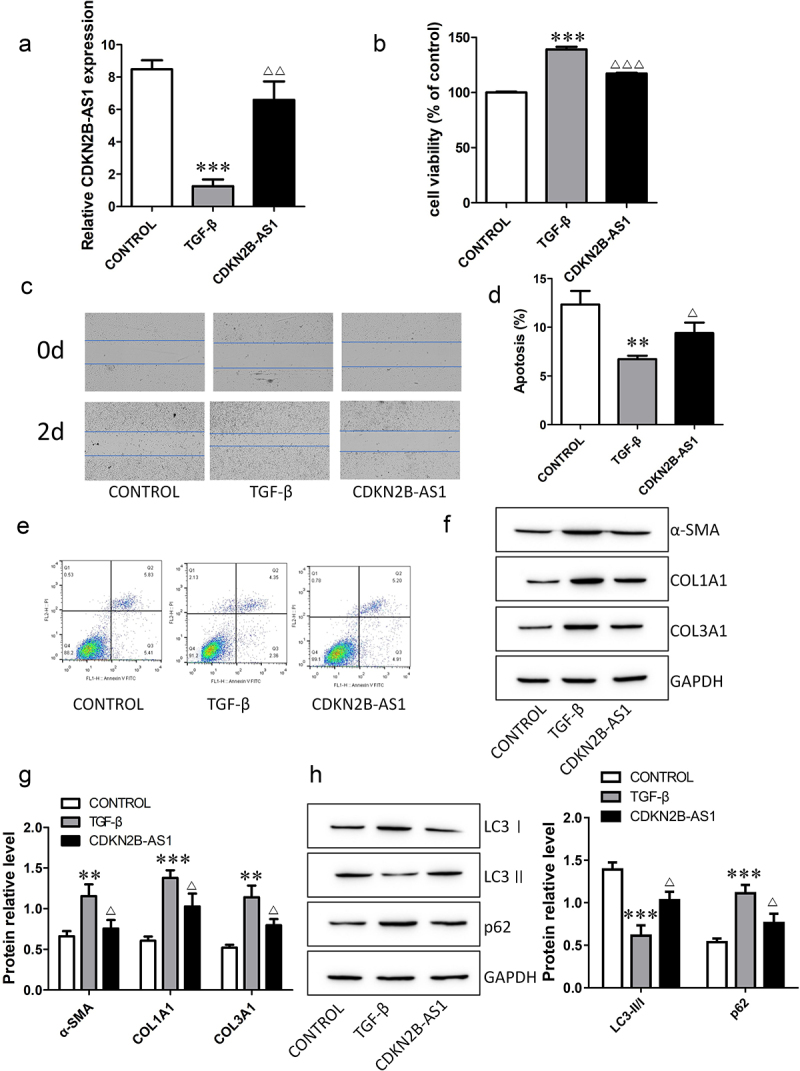
Overexpression of CDKN2B-AS1 alleviates TGF-β-induced fibroblast activation. a: The expression of CDKN2B-AS1 was determined by RT–qPCR. b: HFL-1 cell viability was analyzed by a CCK-8 assay and compared with that under TGF-β treatment. c: The HFL-1 cell migration ability was evaluated by a wound healing assay. d, e: Flow cytometric detection of HFL-1 cell apoptosis. f, g: Western blot verification of α-SMA, COL1A1, and COL3A1 protein expression levels. h: Western blot verification of LC3I, LC3II, and p62 protein expression levels. ***p < 0.001 and **p < 0.01 compared with the CONTROL group; ***p < 0.001, **p < 0.01, and *p < 0.05 compared with the TGF-β group. TGF-β: TGF-β-induced group, CDKN2B-AS1: TGF-β-induced with CDKN2B-AS1-overexpression group. Data are presented as means ± SD (n = 3).

To investigate the functions of CDKN2B-AS1 in IPF, we transfected the CDKN2B-AS1 overexpression plasmid into TGF-β-induced cells. The expression of CDKN2B-AS1 decreased after TGF-β induction, and the level of CDKN2B-AS1 increased after overexpression of CDKN2B-AS1 (Figure 2a). CCK-8 and wound healing assays confirmed that CDKN2B-AS1 significantly inhibited cell proliferation and migration and reversed the effect of TGF-β (Figure 2b,c). Cell apoptosis decreased after TGF-β treatment, while overexpression of CDKN2B-AS1 increased cell apoptosis (Figure 2d,e). We also analyzed the expression levels of fibrosis-associated proteins. CDKN2B-AS1 abolished the activation of fibroblasts treated with TGF-β and decreased the expression of α-SMA, COL1A1 and COL3A1 (Figure 2f,G). Moreover, we evaluated the changes in the autophagy-related proteins LC3I, LC3II and p62. Overexpression of CDKN2B-AS1 increased LC3II/LC3I and decreased p62 (Figure 2h). Our results show that CDKN2B-AS1 overexpression inhibited the activation of fibroblasts and acted through autophagy.

### Overexpression of CDKN2B-AS1 relieves bleomycin-induced pulmonary fibrosis in mice

**Figure 3.**
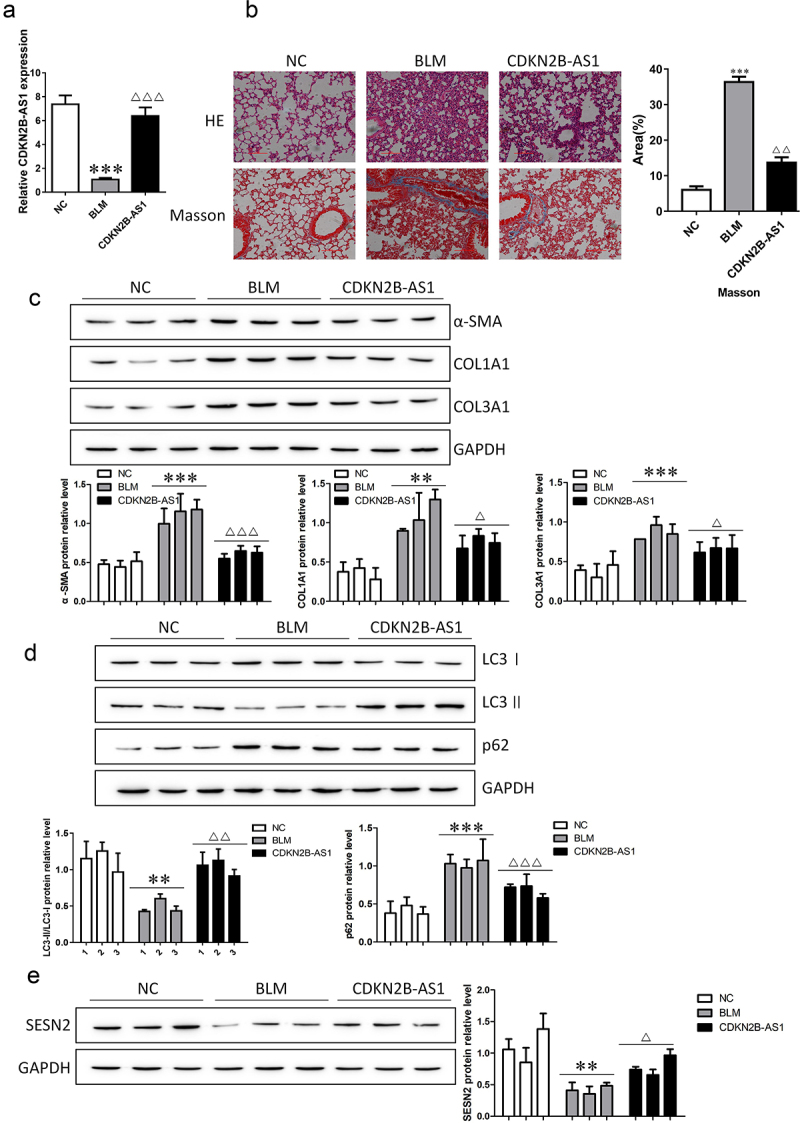
Increasing CDKN2B-AS1 mitigated pulmonary fibrosis under BLM treatment. a: RT–qPCR detection of CDKN2B-AS1 expression in mice. b: HE and Masson staining were used to discover the effect of CDKN2B-AS1 on BLM-induced lung fibrosis in mice. c: α-SMA, COL1A1, and COL3A1 protein expression levels were measured by Western blotting. d: Western blotting verification of LC3I, LC3II, and p62 protein expression. e: The expression level of the SESN2 protein was detected by Western blotting. ***p < 0.001 and **p < 0.01 compared with the NC group; ***p < 0.001, **p < 0.01, and *p < 0.05 compared with the BLM group. NC: negative control group, BLM: BLM-induced group, and CDKN2B-AS1: BLM-induced with CDKN2B-AS1-overexpression group. Data are presented as means ± SD (n = 3).

We verified whether CNKN2B plays a role in pulmonary fibrosis in mice. After bleomycin (BLM) treatment, adenovirus carrying CNKN2B was injected, and CNKN2B expression was detected. The expression level of CNKN2B was decreased after BLM induction, and the expression level of CNKN2B was increased after adenovirus injection (Figure 3a). The results of HE and Masson staining illustrated that BLM induced significant thickening of the alveolar septum, which was filled with fibrous tissue, and the deposition of collagen fibers increased; thus, overexpression of CNKN2B extenuated lung fibrosis in mice (Figure 3b). We examined the changes in the fibrosis-related proteins α-SMA, COL1A1, and COL3A1 and the autophagy-related proteins LC3I, LC3II, and p62. CNKN2B reversed the BLM-induced up-regulation of fibrosis-related proteins α-SMA, COL1A1 in α-SMA, COL1A1, COL3A1, up-regulation of autophagy associated protein p62 and down-regulation of LC3II/LC3I. In addition, the expression level of SESN2 decreased in BLM-induced mice, and SESN2 was upregulated after CDKN2B-AS1 overexpression. Our results suggest that the upregulation of CDKN2B-AS1 inhibits the development of lung fibrosis in mice.

### CDKN2B-AS1 is the sponge of miR-199a-5p

**Figure 4.**
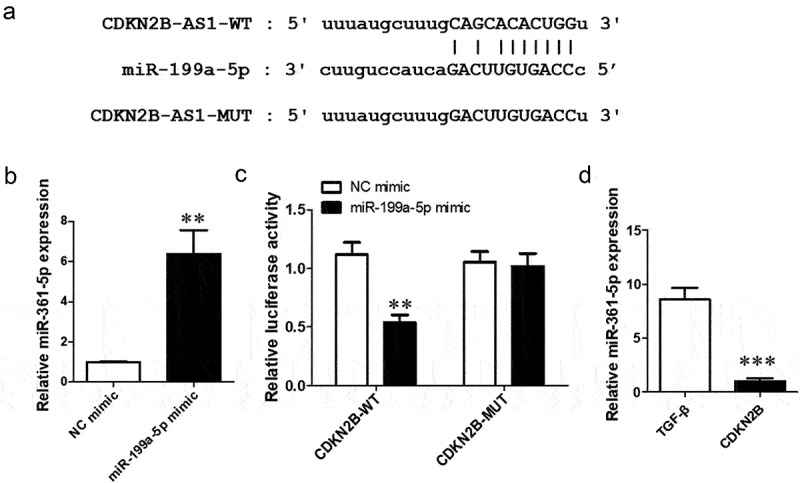
miR-199a-5p is the target miRNA of CDKN2B-AS1. a: CDKN2B-AS1 and miR-199a-5p binding site sequences, including the CDKN2B-AS1 wild type (WT) and mutant type (MUT). b: The expression of miR-199a-5p was examined by RT–qPCR; **p < 0.01 compared with the NC mimic. c: The targeting relationship between CDKN2B-AS1 and miR-199a-5p was confirmed by a dual luciferase reporter assay. **p < 0.01 compared with the contransfection CDKN2B-AS1-WT and the NC mimic. d: RT–qPCR was used to detect the level of miR-199a-5p after overexpression of CDKN2B-AS1. ***p < 0.001 compared with the TGF-β group. Data are presented as means ± SD (n = 3).

To discover the mechanism of action of CDKN2B-AS1 in pulmonary fibrosis, we analyzed the relationship between CDNK2B and miRNA by using the bioinformatics website ‘StarBase’ and found that a binding site between CDNK2B and miR-199a-5p exists (Figure 4a), which means that miR-199a is the target of CDNK2B. RT–qPCR was used to detect the function of the miR-199a-5p mimic, and the expression of miR-199a-5p increased significantly after transfection of the miR-199a-5p mimic (Figure 4b). The dual luciferase reporter assay confirmed that luciferase activity was decreased with the cotrensfection of the CDKN2B-AS1 wild-type (WT) and miR-199a-5p mimic compared with the cotransfection of the CDKN2B-AS1 wild-type (WT) and negative control (NC) mimic (Figure 4c). In addition, after overexpression of CDKN2B-AS1, the level of miR-199a-5p was obviously reduced (Figure 4d). This evidence indicates that CDKN2B-AS1 can be used as a sponge for miR-199a-5p.

### CDKN2B-AS1 negatively regulates the profibrotic function of miR-199a-5p

**Figure 5.**
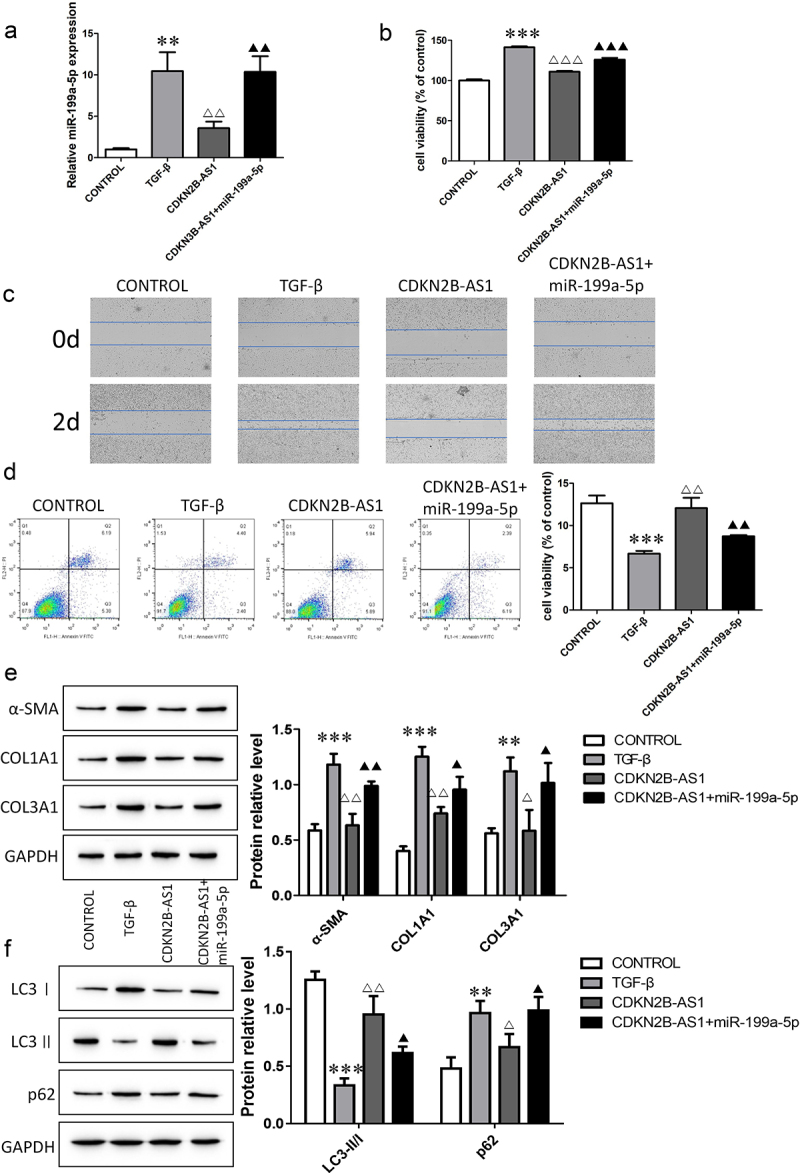
The CDKN2B-AS1/miR-199a-5p axis regulates the evolution of fibrosis. a: RT–qPCR was used to determine the expression level of miR-199a-5p. b, c: CCK-8 and wound healing assay detection of cell proliferation and migration ability. d: Flow cytometric analysis of cell apoptosis. e, f: The protein expression levels of α-SMA, COL1A1, COL3A1, LC3I, LC3II, and p62 were determined by Western blotting. ***p < 0.001 and **p < 0.01 compared with the CONTROL group; ***p < 0.001, **p < 0.01, and *p < 0.05 compared with the TGF-β group; ***p < 0.001, **p < 0.01, and *p < 0.05, compared with the CDKN2B-AS1 group. TGF-β: TGF-β-induced group, CDKN2B-AS1: TGF-β-induced with CDKN2B-AS1-overexpression group, and CDKN2B-AS1+ miR-199a-5p: TGF-β-induced with CDKN2B-AS1- and miR-199a-5p-overexpression group. Data are presented as means ± SD (n = 3).

We transfected miR-199a-5p mimics on the basis of CDKN2B-AS1 overexpression in TGF-β-induced HFL-1 cells. The detection of the expression of miR-199a-5p indicated that the level of miR-199a-5p increased after TGF induction; the level of miR-199a-5p decreased after overexpression of CDKN2B-AS1; and the level of miR-199a- 5p was restored after transfection of the miR-199a-5p mimic (Figure 5a). Cell proliferation and migration were measured by CCK-8 and wound healing assays, and the results showed that miR-199a-5p promoted cell proliferation and migration (Figure 5b,c). Cell apoptosis also changed significantly, and fibroblast apoptosis decreased after transfection with the miR-199a-5p mimic (Figure 5d). The detection of α-SMA, COL1A1, and COL3A1 protein expression showed that miR-199a-5p promoted α-SMA, COL1A1, and COL3A1 levels (Figure 5e). The detection of LC3I, LC3II, and p62 protein expression showed that miR-199a-5p decreased LC3II/LC3I levels and increased p62 levels (Figure 5f). Our findings demonstrate that miR-199a-5p promotes fibrosis and is negatively regulated by CDKN2B-AS1.

### miR-199a-5p negatively regulates SESN2 expression

**Figure 6.**
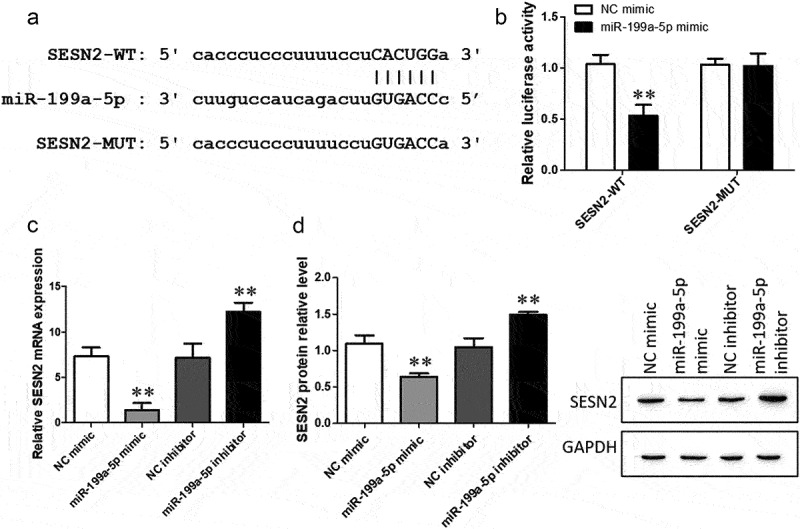
SESN2 is the target gene of CDKN2B-AS1 and its site of interaction. a: SESN2 and miR-199a-5p binding site sequences, including SESN2-WT and MUT. b: The targeting relationship between SESN2 and miR-199a-5p was detected by a dual luciferase reporter assay. **p < 0.01 compared with the cotransfection of SESN2-WT and the NC mimic. c: RT–qPCR was used to determine the mRNA expression level of SESN2. d: The expression of SESN2 was determined by Western blotting. **p < 0.01 compare with the NC group. Data are presented as means ± SD (n = 3).

Furthermore, ‘StarBase’ predicted that the miR-199a-5p target gene is SESN2, which is related to the regulation of autophagy. The binding sites of miR-199a-5p and SESN2 are shown in Figure 6a. Next, the dual luciferase reporter gene assay showed that luciferase activity under the SESN2-WT and miR-199a-5p mimic combination was lower than that under the combination of SESN2-WT and the NC mimic, while no differences were found in the group that received the combination of SESN2-MUT and the miR-199a-5p/NC mimic (Figure 6b). The effects of the miR-199a-5p mimic and inhibitor on the expression of SESN2 mRNA and protein were detected by RT–qPCR and Western blotting. The miR-199a-5p mimic inhibited the expression level of SESN2, while the miR-199a-5p inhibitor promoted the level of SESN2 (Figure 6c,d). Our results reveal that miR-199a-5p inhibits SESN2 expression.

### miR-199a-5p regulates fibrogenesis by directly targeting the expression of SESN2

**Figure 7.**
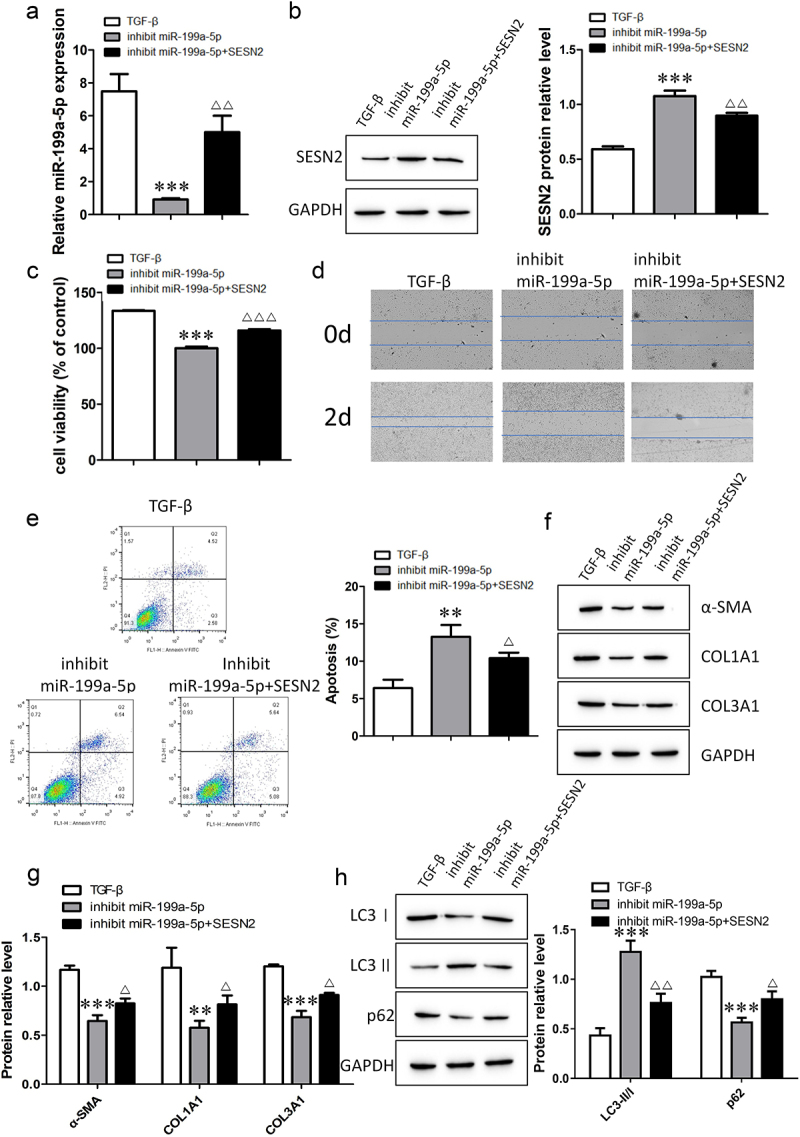
The miR-199a-5p/SESN2 axis regulates fibrogenesis via autophagy. a: The expression of miR-199a-5p was determined by RT–qPCR. b: The expression level of SESN2 protein was detected by Western blotting. c: Cell viability was analyzed by a CCK-8 assay. d: Cell migration ability was evaluated by a wound healing assay. e: Flow cytometric detection of cell apoptosis. f- h: Western blotting verification of α-SMA, COL1A1, COL3A1, LC3I, LC3II, and p62 protein expression levels. ***p < 0.001 and **p < 0.01 compared with the TGF-β group; ***p < 0.001, **p < 0.01, and *p < 0.05 compared with the miR-199a-5p-inhibition group. TGF-β: TGF-β-induced group, inhibit miR-199a-5p: TGF-β-induced with miR-199a-5p-inhibition group, inhibit miR-199a-5p+SESN2: TGF-β-induced with miR-199a-5p- and SESN2-inhibition group. Data are presented as means ± SD (n = 3).

To understand the mechanism by which miR-199a-5p and SESN2 are involved in pulmonary fibrosis, we investigated the behavioral transformation of TGF-β-induced cells by inhibiting miR-199a-5p and SESN2. The RT–qPCR results showed that the expression of miR-199a-5p was downregulated after transfection with the miR-199a-5p inhibitor, and the expression of miR-199a-5p was upregulated after cotransfection with the miR-199a-5p inhibitor and SESN2 inhibitor (Figure 7a). Measurement of the expression of SESN2 protein indicated that the level of SESN2 increased after miR-199a-5p inibition, and the level of SESN2 decreased after simultaneous miR-199a-5p and SESN2 inhibition (Figure 7b). The detection of cell viability, migration and apoptosis revealed that cell viability and migration ability decreased and apoptosis increased with the inhibition of miR-199a-5p; moreover, the inhibition of miR-199a-5p and SESN2 restored cell viability and migration ability, and apoptosis was subsequently reduced (Figure 7c-e). Western blot analysis was used to determine the protein expression of α-SMA, COL1A1, COL3A1, LC3I, LC3II, and p62. We discovered that inhibiting SESN2 abolished the suppression of fibrosis-related proteins and autophagy-related proteins by miR-199a-5p. Together, these findings suggest that miR-199a-5p is involved in autophagy and the regulation of fibrogenesis through SESN2.

## Discussion and conclusion

In this study, we found that CDKN2B-AS1 was expressed at low levels in TGF-β-induced cells and BLM-induced mice. Overexpression of CDKN2B-AS1 inhibited cell proliferation and migration and promoted cell apoptosis and autophagy. In addition, overexpression of CDKN2B-AS1 significantly alleviated lung fibrosis in BLM-treated mice. More importantly, CDKN2B-AS1 acts as a sponge of miR-199a-5p to regulate the expression of SESN2, thereby affecting fibrogenesis. It is worth noting that SESN2 is involved in the positive regulation of autophagy, and SESN2 is a target gene of miR-199a-5p. These results suggest that autophagy activity mediates the development and progression of pulmonary fibrosis. In general, after CDKN2B-AS1 overexpression, pulmonary fibrosis is improved and autophagy is inhibited via the miR-199a-5p/SESN2 axis, which provides a new direction for the diagnosis and treatment of IPF.

Cumulative studies [28,29] have shown the importance of the abnormal expression or function of lncRNAs in the development of fibrotic diseases. For example, the expression of lncRNA DNM3OS is upregulated in PF cells, and DNM3OS can specifically regulate the expression of miR-199a-3p/5p and miR-214-3p, affect SMAD and non-SMAD components in TGF-β signaling, and then participate in PF progression [30]. lncRNA H19 is highly expressed in the lung tissues and cells of PF rats. Knockdown of H19 can significantly inhibit the expression of α-SMA and collagen I and III in HBE and A549 cells induced by TGF-β1 and inhibit PF in vitro [31]. CDKN2B-AS1 is a newly identified lncRNA that is related to many types of diseases. However, the underlying mechanism by which CDKN2B-AS1 participates in the regulation of IPF needs further investigation. We determined that CDKN2B-AS1 expression was decreased compared with that in control cells. Similarly, CDKN2B-AS1 expression was decreased in mice with IPF. Therefore, we speculated that CDKN2B-AS1 may be a major element in the development of IPF.

lncRNAs regulate the expression of downstream miRNAs, and the dysregulation of miRNAs has been shown to be related to a variety of lung diseases [32], including lung cancer, asthma and PF. The expression of lncRNA ATB is increased in TGF-β-induced cells, and ATB downregulates miR-200c, promotes the expression of ZEB1, and then promote the processes of epithelial-mesenchymal transition (EMT) and renal fibrosis [33]. Ligustrazin upregulates the expression of miR-193a, inhibits the activation of the PI3K/AKT/mTOR signaling pathway, promotes autophagy in lung epithelial cells, and relieves paraquat-induced PF [34]. We discovered the targeting relationship between CDKN2B-AS1 and miR-199a-5p by using a bioinformatics database. miR-199a-5p is highly expressed in TGF-induced cells, which is consistent with the findings of Lino Cardenas et al [35]. The results of overexpression of CDKN2B-AS1 and miR-199a-5p indicate that CDKN2B-AS1 negatively regulates the fibrosis-promoting function of miR-199a-5p.

Previous studies [36] have revealed that autophagy is a significant link in PF. Hill et al [20]. reported that the inhibition of autophagy promotes the epithelial–mesenchymal transition and fibroblast differentiation. Liu et al. [37] verified that the increased expression of BBC3 in macrophages promotes silica-induced autophagy and the proliferation and migration of fibroblasts, thereby accelerating the development of PF. We found that miR-199a-5p inhibits SESN2 expression. Perhaps more importantly, SESN2 participates in autophagy with a positive regulatory effect, and siRNA interference with SESN2 can inhibit autophagy induced by a variety of stimuli [38]. The role of SESN2 in IPF exerted through autophagy regulation remains elusive. In this study, we have verified the molecular mechanism of CDKN2B-AS1 alleviating idiopathic pulmonary fibrosis by regulating the miR-199a-5p/SESN2 molecular axis in animal experiments and cell experiments, but there is still a shortcoming that there is no clinical application to further verify our proposed molecular mechanism, follow-up research will be carried out in this direction, so that our research can be practically applied to the clinical treatment of idiopathic pulmonary fibrosis.

### Conclusions

We found that the expression of CDKN2B-AS1 was decreased in TGF-β-treated cells and BLM-treated mice. In the end, we demonstrated that overexpression of CDKN2B-AS1 targets down-regulation of miR-199a-5p, facilitated the expression of SESN2 and induced autophagy, thus inhibits IPF.

## References

[1] Wolters PJ, Collard HR, Jones KD. Pathogenesis of idiopathic pulmonary fibrosis. Annu Rev Pathol. 2014;9(1):157–179. 24050627 10.1146/annurev-pathol-012513-104706 PMC4116429

[2] Liu YM, Nepali K, Liou JP. Idiopathic pulmonary fibrosis: current status, recent progress, and emerging targets. J Med Chem. 2017;60(2):527–553. 28122457 10.1021/acs.jmedchem.6b00935

[3] King T, Bradford WZ, Castro-Bernardini S, et al. A phase 3 trial of pirfenidone in patients with idiopathic pulmonary fibrosis. N Engl J Med. 2014;370(22):2083–2092. 24836312 10.1056/NEJMoa1402582

[4] Kropski J. Biomarkers and early treatment of idiopathic pulmonary fibrosis. Lancet Respir Med. 2019;7(9):725–727. 31326318 10.1016/S2213-2600(19)30256-5 PMC6839826

[5] Mathy NW, Chen XM. Long non-coding RNAs (lncRNAs) and their transcriptional control of inflammatory responses. J Biol Chem. 2017;292(30):12375–12382. 28615453 10.1074/jbc.R116.760884 PMC5535013

[6] Wang L, Zhang N, Zhang, Y, et al. Landscape of transcription and long non-coding RNAs reveals new insights into the inflammatory and fibrotic response following ventilator-induced lung injury. Respir Res. 2018;19(1):122. 29929510 10.1186/s12931-018-0822-z PMC6013938

[7] Cai H, Zheng Y, Wen Z, et al. LncRNA AIRN influences the proliferation and apoptosis of hepatocellular carcinoma cells by regulating STAT1 ubiquitination. Arch Pharm Res. 2021;44(4):414–426. 33759138 10.1007/s12272-021-01317-7

[8] Song C, Qi, Y, Zhang, J, et al. CDKN2B-AS1: an indispensable long non-coding RNA in multiple diseases. Curr Pharm Des. 2020;26(41):5335–5346. 32767927 10.2174/1381612826666200806102424

[9] Wang G, Xu G, Wang W. Long noncoding RNA CDKN2B-AS1 facilitates lung cancer development through regulating miR-378b/NR2C2. Onco Targets Ther. 2020;13:10641–10649. 33116641 10.2147/OTT.S261973 PMC7585785

[10] Chen X, Yu X, Shen E. Overexpression of CDKN2B is involved in poor gastric cancer prognosis. J Cell Biochem. 2019;120(12):19825–19831. 31297846 10.1002/jcb.29287

[11] Li Y, Zheng -L-L, Huang D-G, et al. LNCRNA CDKN2B-AS1 regulates mesangial cell proliferation and extracellular matrix accumulation via miR-424-5p/HMGA2 axis. Biomed Pharmacother. 2020;121:109622. 31707340 10.1016/j.biopha.2019.109622

[12] Ou M, Li X, Zhao S, et al. Long non-coding RNA CDKN2B-AS1 contributes to atherosclerotic plaque formation by forming RNA-DNA triplex in the CDKN2B promoter. EBioMedicine. 2020;55:102694. 32335370 10.1016/j.ebiom.2020.102694 PMC7184162

[13] Du Y, Hao X, Liu X. Low expression of long noncoding RNA CDKN2B-AS1 in patients with idiopathic pulmonary fibrosis predicts lung cancer by regulating the p53-signaling pathway. Oncol Lett. 2018. DOI:10.3892/ol.2018.7910 PMC5835920 29541247

[14] Lederer D, Martinez F, Longo DL. Idiopathic pulmonary fibrosis. N Engl J Med. 2018;378(19):1811–1823. 29742380 10.1056/NEJMra1705751

[15] Heukels P, Moor CC, von der Thüsen JH, et al. Inflammation and immunity in IPF pathogenesis and treatment. Respir Med. 2019;147:79–91. 30704705 10.1016/j.rmed.2018.12.015

[16] Yuan H, Xu J, Zhu Y, et al. Activation of calcium‑sensing receptor‑mediated autophagy in high glucose‑induced cardiac fibrosis in vitro. Mol Med Rep. 2020;22(3):2021–2031. 32705187 10.3892/mmr.2020.11277 PMC7411369

[17] Racanelli AC, Choi AMK, Choi ME. Autophagy in chronic lung disease. Prog Mol Biol Transl Sci. 2020;172:135–156. 32620240 10.1016/bs.pmbts.2020.02.001 PMC8608369

[18] Li Y, Liu R, Wu J, et al. Self-eating: friend or foe? The emerging role of autophagy in fibrotic diseases. Theranostics. 2020;10(18):7993–8017. 32724454 10.7150/thno.47826 PMC7381749

[19] Sosulski M, Gongora R, Danchuk S, et al. Deregulation of selective autophagy during aging and pulmonary fibrosis: the role of TGF β1. Aging Cell. 2015;14(5):774–783. 26059457 10.1111/acel.12357 PMC4568965

[20] Hill C, Li, J, Liu, D, et al. Autophagy inhibition-mediated epithelial-mesenchymal transition augments local myofibroblast differentiation in pulmonary fibrosis. Cell Death Dis. 2019;10(8):591. 31391462 10.1038/s41419-019-1820-x PMC6685977

[21] Lin X, Bai, D, Wei, Z, et al. Curcumin attenuates oxidative stress in RAW264.7 cells by increasing the activity of antioxidant enzymes and activating the Nrf2-Keap1 pathway. PLOS ONE. 2019;14 (5):e0216711. 31112588 10.1371/journal.pone.0216711 PMC6528975

[22] Liu P, Feng Y, Dong D, et al. Enhanced renoprotective effect of IGF-1 modified human umbilical cord-derived mesenchymal stem cells on gentamicin-induced acute kidney injury. Sci Rep. 2016;6(1):20287. 26830766 10.1038/srep20287 PMC4735814

[23] Zhao L, Feng Y, Chen X, et al. Effects of IGF-1 on neural differentiation of human umbilical cord derived mesenchymal stem cells. Life Sci. 2016;151:93–101. 26946309 10.1016/j.lfs.2016.03.001

[24] Ding LZ, Teng X, Zhang Z-B, et al. Mangiferin inhibits apoptosis and oxidative stress via BMP2/Smad-1 signaling in dexamethasone-induced MC3T3-E1 cells. Int J Mol Med. 2018. DOI:10.3892/ijmm.2018.3506. PMC5846652 29484386

[25] Guo Y, Zhu X, Sun X. COTI-2 induces cell apoptosis in pediatric acute lymphoblastic leukemia via upregulation of miR-203. Bioengineered. 2020;11(1):201–208. 32063077 10.1080/21655979.2020.1729927 PMC7039633

[26] Li J, Li, P, Zhang, G, et al. CircRNA TADA2A relieves idiopathic pulmonary fibrosis by inhibiting proliferation and activation of fibroblasts. Cell Death Dis. 2020;11(7):553. 32694556 10.1038/s41419-020-02747-9 PMC7374112

[27] N K, Kamio K, Azuma A, et al. *Exosome-derivedmicroRNA-22* ameliorates pulmonary fibrosis by regulating fibroblast-to-myofibroblast differentiation *in vitro* and *in vivo*. J Nippon Med Sch. 2020;87(3):118–128. 31776321 10.1272/jnms.JNMS.2020_87-302

[28] Ayana R, Singh S, Pati S. Decoding crucial LncRNAs implicated in neurogenesis and neurological disorders. Stem Cells Dev. 2017;26(8):541–553. 28095733 10.1089/scd.2016.0290

[29] Yang M, Wang, M, Li, X, et al. The role of lncRNAs in signaling pathway implicated in CC. J Cell Biochem. 2019;120(3):2703–2712. 30552693 10.1002/jcb.26835

[30] Savary G, Dewaeles, E, Diazzi, S, et al. The long noncoding RNA DNM3OS is a reservoir of fibromirs with major functions in lung fibroblast response to TGF-β and pulmonary fibrosis. Am J Respir Crit Care Med. 2019;200(2):184–198. 30964696 10.1164/rccm.201807-1237OC

[31] Wang X, Cheng Z, Dai L, et al. Knockdown of long noncoding RNA H19 represses the progress of pulmonary fibrosis through the transforming growth factor β/Smad3 pathway by regulating MicroRNA 140. Mol Cell Biol. 2019;39(12). DOI:10.1128/MCB.00143-19. PMC6549464 30988156

[32] Zhen Q, Gao L-N, Wang R-F, et al. LncRNA DANCR promotes lung cancer by sequestering miR-216a. Cancer Control. 2018;25(1):1073274818769849. 29651883 10.1177/1073274818769849 PMC6852365

[33] Liu Y, Li Y, Xu Q, et al. Long non-coding RNA-ATB promotes EMT during silica-induced pulmonary fibrosis by competitively binding miR-200c. Biochim Biophys Acta Mol Basis Dis. 2018;1864(2):420–431. 29113749 10.1016/j.bbadis.2017.11.003

[34] Liu MW, Su, MX, Tang, DY, et al. Ligustrazin increases lung cell autophagy and ameliorates paraquat-induced pulmonary fibrosis by inhibiting PI3K/Akt/mTOR and hedgehog signalling via increasing miR-193a expression. BMC Pulm Med. 2019;19(1):35. 30744607 10.1186/s12890-019-0799-5 PMC6371511

[35] Lino Cardenas CL, Henaoui IS, Courcot E, et al. miR-199a-5p is upregulated during fibrogenic response to tissue injury and mediates TGFbeta-induced lung fibroblast activation by targeting caveolin-1. PLoS Genet. 2013;9(2):e1003291. 23459460 10.1371/journal.pgen.1003291 PMC3573122

[36] Lv X, Li K, Hu Z. Autophagy and pulmonary fibrosis. Adv Exp Med Biol. 2020;1207:569–579. 32671775 10.1007/978-981-15-4272-5_40

[37] Liu H, Cheng Y, Yang J, et al. BBC3 in macrophages promoted pulmonary fibrosis development through inducing autophagy during silicosis. Cell Death Dis. 2017;8(3):e2657. 28277537 10.1038/cddis.2017.78 PMC5386570

[38] Ambrosio S, Saccà CD, Amente S, et al. Lysine-specific demethylase LSD1 regulates autophagy in neuroblastoma through SESN2-dependent pathway. Oncogene. 2017;36(48):6701–6711. 28783174 10.1038/onc.2017.267 PMC5717079

